# Zebrafish as a Model for Obesity and Diabetes

**DOI:** 10.3389/fcell.2018.00091

**Published:** 2018-08-20

**Authors:** Liqing Zang, Lisette A. Maddison, Wenbiao Chen

**Affiliations:** ^1^Department of Molecular Physiology and Biophysics, Vanderbilt University School of Medicine, Nashville, TN, United States; ^2^Graduate School of Regional Innovation Studies, Mie University, Tsu, Japan; ^3^Center for Reproductive Biology, Washington State University, Pullman, WA, United States

**Keywords:** zebrafish, obesity, diabetes, transgenic models, disease models, animal

## Abstract

Obesity and diabetes now considered global epidemics. The prevalence rates of diabetes are increasing in parallel with the rates of obesity and the strong connection between these two diseases has been coined as “diabesity.” The health risks of overweight or obesity include Type 2 diabetes mellitus (T2DM), coronary heart disease and cancer of numerous organs. Both obesity and diabetes are complex diseases that involve the interaction of genetics and environmental factors. The underlying pathogenesis of obesity and diabetes are not well understood and further research is needed for pharmacological and surgical management. Consequently, the use of animal models of obesity and/or diabetes is important for both improving the understanding of these diseases and to identify and develop effective treatments. Zebrafish is an attractive model system for studying metabolic diseases because of the functional conservation in lipid metabolism, adipose biology, pancreas structure, and glucose homeostasis. It is also suited for identification of novel targets associated with the risk and treatment of obesity and diabetes in humans. In this review, we highlight studies using zebrafish to model metabolic diseases, and discuss the advantages and disadvantages of studying pathologies associated with obesity and diabetes in zebrafish.

## Introduction

The prevalence of overweight and obesity has steadily increased worldwide in the past several decades. In 2016, more than 1.9 billion adults were overweight, and of these over 650 million were obese (WHO, 2017). This is primarily due to excess food consumption (Vandevijvere et al., 2015). Overweight and obesity are major risk factors for numerous chronic diseases, including cardiovascular diseases, diabetes, and certain types of cancer (Haslam and James, 2005). In the United States, class III obese individuals (BMI ≥ 40 kg/m^2^) have a six-fold increase in diabetes risk over normal-weight individuals (Leung et al., 2017) and more than 90% of people with type 2 diabetes mellitus (T2DM) are overweight or obese. The global increase of overweight and obesity largely explains the incidence and prevalence of type 2 diabetes over the past 20 years. Obesity and T2DM can substantially decrease life expectancy, diminish quality of life, and impose a large economic burden to society (Leung et al., 2017).

Both obesity and T2DM have high heritability (Poulsen et al., 2001; Willemsen et al., 2015). Recent genome wide association studies and whole exome sequencing studies have identified a large number of genetic variants that are associated with overweight/obesity and/or T2DM (Lawlor et al., 2017; Loos, 2018). In most cases, however, the causative genes for these linked variants are uncertain and the mechanism by which these variants contribute to the disease phenotypes is unclear (Loos, 2018). Furthermore, the aggregate effect of all the variants only account for a small fraction of the heritability of these conditions (Fuchsberger et al., 2016). It is likely that more alleles are yet to be discovered to play a role in obesity and T2DM susceptibility.

The wealth of human genetic and epidemiological data on obesity and T2DM provides ample opportunity for mechanistic investigations in animal models. Zebrafish is a well-established model system for developmental biology, human genetics, and human diseases (Dooley and Zon, 2000; Gibert et al., 2013; Freifeld et al., 2017). Several features have propelled zebrafish to its current prominence in developmental biology and disease modeling. It is a vertebrate, having high degree of genetic, anatomical, and physiological similarities to humans. It is fecund, easy to maintain in large number and has a relative short generation time, allowing facile genetic, and chemical genetic screens (Kimmel et al., 1995; MacRae and Peterson, 2015). Its external development affords easy accessibility to embryonic and genetic manipulations (Kimura et al., 2014; Hoshijima et al., 2016; Yin et al., 2016). The optical transparency of its embryos permits time lapse live imaging (Hall et al., 2009; Herrgen et al., 2009; Feierstein et al., 2015). Although traditionally used for developmental biology, zebrafish has recently been used to investigate metabolic diseases. Here, we will review some of the recent studies using zebrafish to model human metabolic diseases, with an emphasis on obesity, and diabetes. We will discuss the advantages and disadvantages of studying pathologies associated with obesity and diabetes in zebrafish.

## Zebrafish obesity models

### Lipid metabolism and adipose biology in zebrafish

Obesity is a consequence of positive energy balance. Regulation of energy intake and expenditure involves many organ systems including the brain, intestines, skeletal muscle, and adipose tissue (Cai, 2013; Dailey, 2014; Periasamy et al., 2017). Therefore, whole animal models are essential for better understanding of the development and progression of metabolic dysfunction. Zebrafish is an excellent model in which to study metabolic dysfunction because they have the key organs that are important for regulation of energy homeostasis and metabolism in mammals, including digestive organs, adipose tissues, and skeletal muscle (Lieschke and Currie, 2007; Schlegel and Stainier, 2007). The key functions such as appetite regulation, insulin regulation and lipid storage are also well conserved (Elo et al., 2007; Flynn et al., 2009; Nishio et al., 2012). Similar to mammals, excess nutrients in zebrafish cause increased plasma triglyceride levels and hepatic steatosis (Oka et al., 2010). Obese zebrafish also exhibit dysregulation of pathways that control lipid metabolism, including SREBF1, PPARs, NR1H3, and LEP (Oka et al., 2010). The conservation of these metabolic pathways that play key roles in adipocyte differentiation, energy homeostasis (Den Broeder et al., 2015), and cholesterol metabolism (Schlombs et al., 2003) demonstrates zebrafish as a suitable model for human lipid metabolism. However, zebrafish is an ectotherm species and its metabolic rate is not regulated by environmental temperature. Consistent with this, zebrafish does not have brown adipocyte tissues (BAT).

A primary characteristic of obesity is adipose hypertrophy and hyperplasia. Zebrafish have multiple adipose tissue depots and their development has been characterized (Flynn et al., 2009). Neutral lipid droplets first appear in visceral adipocytes and accumulate as zebrafish grow. Similar to mammalian white adipose tissue (WAT), early-stage zebrafish adipocytes contain multiple small lipid droplets while mature zebrafish adipocytes have a single large lipid droplet. As occurs in mammals, the adipocyte lineage expresses *pparg*, and *fabp4* (Flynn et al., 2009). Visceral adiposity is a critical risk factor for T2DM and other metabolic diseases (Ahima and Lazar, 2013). In zebrafish, like in mammals, lipids are stored in visceral, intramuscular and subcutaneous adipocyte depots (Song and Cone, 2007), providing the opportunity to understand the regulation of body fat distribution. The high degree of conservation in distribution and formation of adipose tissue in the zebrafish compared to mammals makes it an appropriate model to study obesity.

### Methods to quantitate adiposity in zebrafish

**Figure 1:**
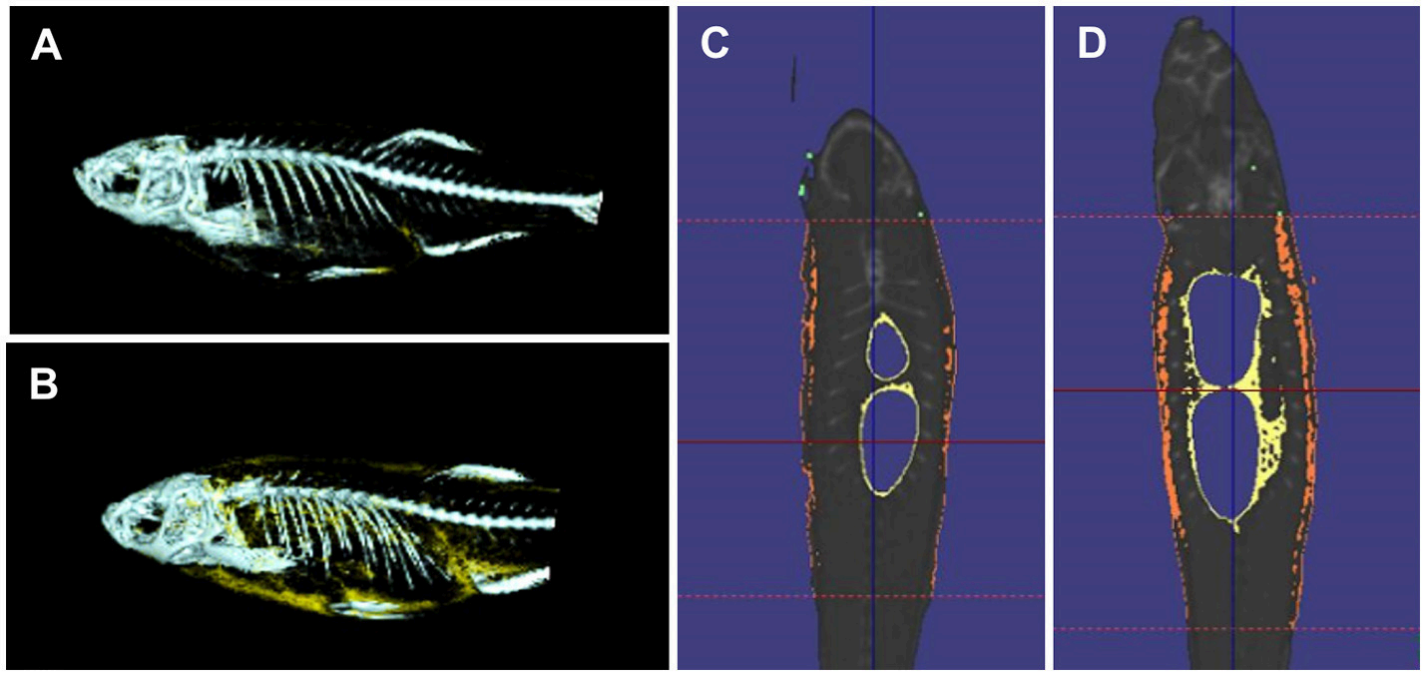
3D micro-CT analysis in normal fed and diet-included obese zebrafish. **(A)** 3D-images of normal fed zebrafish. Gray color indicates skeleton and yellow color means adipocyte tissue. **(B)** 3D-images obese zebrafish. **(C)** Cross-sectional images of normal fed zebrafish. Yelloe color indicates visceral adipose tissue and orange color indicates subcutaneous adipocyte tissue. **(D)** Cross-sectional images of obese zebrafish.

Quantitative measures of adiposity are important to assess the degree of obesity-related metabolic derangements. Body mass index (BMI) and quantitative computed tomography (CT) are widely used measurements of adiposity in humans but are more difficult to apply in zebrafish. Commonly used lipophilic dyes for visualizing lipids in histological sections and cultured cells, including Nile red, Oil red O, and Sudan black B, have been utilized to detect lipids in adult zebrafish sections and fixed zebrafish larvae (Marza et al., 2005; Schlegel and Stainier, 2006). With the optical transparency of zebrafish larvae, live-imaging, and fluorescence based screens have been developed for the study of digestive physiology or lipid metabolism. In particular, Nile red has been used for live imaging and quantification of intracellular neutral lipid droplets (Greenspan et al., 1985) as well as for purification of adipocyte tissues (Jones et al., 2008; Flynn et al., 2009; Oka et al., 2010). In addition, a variety of fluorescent lipid analogs and tracers are available, including BODIPY Fatty Acid Analogs, BODIPY-cholesterol analogs and fluorescence reports like PED6, for tracking the metabolism and distribution of exogenous lipids in live zebrafish (Hölttä-Vuori et al., 2010; Anderson et al., 2011). 3D micro-CT is also available for this small animal and allows volume measurement of total adipocyte tissue as well as different fat depots (Hasumura et al., 2012; Figure 1). Recently, Landgraf et al. compared the methodology of quantify zebrafish body fat mass using MR images (MRI) and EchoMRI 4in1 (EchoMRI™; Landgraf et al., 2017). The body fat mass of 8 adult male zebrafish was measured using the two methods and the two techniques showed high correlation. Overall, these methods provide accurate measurements of zebrafish adiposity and provide means for longitudinal monitoring.

A number of obesity models have been developed in zebrafish using diet and genetic manipulations (Table 1). The next section describes the details of these models.

**Table 1:** Zebrafish obesity models.

**Type of model**		**Treatment or genetic manipulation**	**Age**	**Characteristics**	**References**
Induced models	High-fat diet	Heavy whipping cream	Larvae	Lipid accumulation in intersegmental vessels; increased whole-larval triacylglycerol (TAG) and apolipoprotein B levels	Schlegel and Stainier, 2006
		Chicken egg yolk	Larvae, juvenile, adult	Hyperlipidemia, increased adipose tissue area and TAG	Tingaud-Sequeira et al., 2011; Zhou et al., 2015; Kopp et al., 2016; den Broeder et al., 2017; Landgraf et al., 2017
		Corn oil and lard	Adult	Increased body fat	Meguro et al., 2015
	Over-nutrition	Artemia	Adult	Increased BMI, hypertriglyceridemia and hepatosteatosis	Oka et al., 2010; Tainaka et al., 2011; Hasumura et al., 2012; Hiramitsu et al., 2014; Zang et al., 2014, 2015a; Meguro et al., 2015; Ran et al., 2017; Meguro and Hasumura, 2018
	Over-nutrition and high-fat diet	Tetramin and vegetable oil	Juvenile, adult	Increased weight gain, cardiovascular overload	Vargas and Vásquez, 2017
		Artemia and egg yolk powder	Adult	Increased body weight, adipose tissue mass, adipocyte hypertrophy, hyperglycemia and hepatosteatosis	Landgraf et al., 2017
Transgenic lines	*Tg(b-actin:AgRP)*	Overexpression of *agrp*	All stages	Increased linear growth, adipocyte hypertrophy	Ahima and Lazar, 2013
	*Tg(-2.5β-Act:mCherry-miR-27b-SP)*	miR-27b depletion	All stages	Hyperlipidemia, hepatic steatosis and increased white adipose tissue mass	Hsu et al., 2017
	*Tg(krt4Hsa.myrAkt1)^*cy*18^*	Overexpression of *akt1*	Adult	Increased BMI, adipocyte hyperplasia, abnormal fat deposition, and glucose intolerance	Chu et al., 2012
Mutant lines	*Foie gras (hi^1532*B*^)*	Mutation in *trappc11*	Larvae	Hepatomegaly and steatosis	Sadler et al., 2005; DeRossi et al., 2016
	*cdipt^*hi*559*Tg*/+^ (hi^559^)*	Mutation in *cdipt*	Larvae	Hepatic steatosis	Thakur et al., 2011
	*harvest moon (hmn^*z*110^)*	Mutation in *gfpt1*	Larvae	Increased whole body TAG and hepatic steatosis	Hugo and Schlegel, 2017
	*vmp1 (7466^*mu*110^)*	Mutation in *vmp1*	larvae	Hepatic steatosis	Kim et al., 2015
	*ducttrip (dtp^*p*14*nb*^)*.	Mutation in *achy*	Larvae	Mitochondrial dysfunction, hepatic steatosis, and disrupted exocrine pancreas	Yee et al., 2005
	*red moon (rmn)*	Mutation in *slc16a6a*	Larvae	Fasting hepatic steatosis	Hugo et al., 2012
	*vizzini*	Mutation in *gh1*	Larvae, adult	Decreased somatic growth, increased accumulation of adipose tissue	McMenamin et al., 2013
	*cyp2r1 mutants*	*cyp2r1* mutations	Adult	Growth retardation, increased adiposity	Peng et al., 2017
	*plxnd1^*fov*01*b*^*	Mutation in *plxnd1*	Adult	Disproportional SAT, altered body fat distribution after high-fat feeding, protected from insulin resistance	Minchin et al., 2015

### High-fat diet/over-nutrition induced obesity

A common approach to induce obesity is excess fat intake. Obese zebrafish can be conveniently produced by overfeeding starting from the onset of feeding at 5 dpf (day post fertilization). This is advantageous over rodent models since diet can only be manipulated after weaning, which is at least 3 weeks after birth. Although early larvae have no WAT (Imrie and Sadler, 2010), lipid droplets accumulate in the blood stream and other measures such as whole-larval triacylglycerol level may be used as an indicator for quantifying obesity progression (Schlegel and Stainier, 2006; Tingaud-Sequeira et al., 2011). Although heavy cream has been used (Schlegel and Stainier, 2006), chicken egg yolk solution is the most used high-fat diet for zebrafish larvae and juveniles (Tingaud-Sequeira et al., 2011; Zhou et al., 2015; Kopp et al., 2016; den Broeder et al., 2017). The high fat diet rapidly increases zebrafish adiposity.

Adult zebrafish have also been used as an obesity model. The first diet-induced obese (DIO) zebrafish model was reported in 2010 (Oka et al., 2010) where adult fish (3.5 months of age) were fed with 60 mg or 5 mg of freshly hatched *Artemia* per day for 8 weeks (150 calories vs. 20 calories). The overfed zebrafish exhibited increased BMI, hypertriglyceridemia and hepatosteatosis compared to the normal fed zebrafish. Male and female zebrafish showed similar responses to over-nutrition treatment. Furthermore, comparative transcriptome analysis of visceral adipose tissue among zebrafish, mouse, rat and human revealed that lipid metabolism networks of zebrafish are similar to those of mammals.

Besides overfeeding with *Artemia*, several other methods have been used to generate obese zebrafish. Meguro et al. developed custom high-fat zebrafish diets containing 20% corn oil or lard. They demonstrated that these high-fat diets make zebrafish obese (Meguro et al., 2015). Similarly, overfeeding the combination of a commercial tropical fish flakes (Tetramin) and 20% crude vegetable oil to zebrafish for 256 days triggers cardiovascular overload (Vargas and Vásquez, 2017).

It is interesting to note that obesity induced by overfeeding normal fat diet differs from that induced by high fat diet. Landgraf et al. compared the metabolic phenotype of obesity induced by overfeeding of a normal fat diet (NFD; *Artemia* cysts, 22% fat) to that by high fat diet (HFD; egg yolk powder, 59% fat). Although both increase adiposity, fish with NFD-induced obesity are metabolically healthy. In contrast, fish with HFD-induced obesity are metabolically unhealthy, with glucose intolerance, fatty liver, and preferential increase of visceral fat (Landgraf et al., 2017). This is consistent with the “obesity paradox” observed in humans. Overweight and obesity are not always associated with insulin resistance, the major driver and precursor of T2DM. In fact, overweight has also been paradoxically associated with lower mortality (Kokkinos et al., 2009; Flegal et al., 2013). This is thought to be due to the difference in the fat distribution pattern. In general, the visceral fat mass is a better predictor for insulin resistance and T2DM than BMI (Lebovitz and Banerji, 2005; InterAct et al., 2012). Therefore, the pathophysiological consequences of different fat depots are also conserved in zebrafish.

### Genetic models of obesity

Obesity is a complex disease that results of an interaction between genetic and environmental factors. Genetically-modified animal models that reflect human obesity pathology are needed for understanding the physiological and genetic basis of obesity and for the development of pharmaceuticals to treat obesity. Genetic models of obesity have been characterized in zebrafish including transgenic lines expressing obesogenic genes or mutants from targeted mutagenesis and genetic screens.

Underscoring the conservation of metabolic regulation, genetic manipulation of pathways that control body weight in mammalian systems also causes obesity in zebrafish. Transgenic zebrafish obesity models are often generated by mimicking existing mammalian models. The central melanocortin system (CMS), including peptides derived from proopiomelanocortin (POMC), their receptors (MC3R and MC4R), and Agouti-related peptide (AgRP), regulates energy homeostasis in zebrafish as it does in mammals (Ringholm et al., 2002; Hansen et al., 2003). Suppression of central melanocortin receptors by ectopic expression of the hair follicle restricted *Agouti* due to chromosomal translocations underlies one of the classical obese mouse models, Agouti Yellow (Bultman et al., 1992; Lu et al., 1994). This led to the identification of the endogenous melanocortin antagonist AgRP, whose transgenic overexpression in brain also causes obesity (Ollmann et al., 1997). In the zebrafish a genetic model of obesity has been developed by overexpressing AgRP [*Tg(b-actin:AgRP)*] (Song and Cone, 2007). These transgenic zebrafish exhibit an increase in body weight, linear growth, visceral adipose accumulation, and total triglycerides in all stages. The increased adiposity results from both hypertrophic and hyperplastic growth of adipocytes (Song and Cone, 2007). This transgenic zebrafish model demonstrates that central regulation of metabolism is conserved. The microRNA miR-27b has been suggested to be a regulatory hub for lipid metabolism by inhibiting the translation of a number of key lipid-metabolism genes (Vickers et al., 2013). Although several lines of evidence from cell culture support a role of miR-27b in lipid metabolism (Karbiener et al., 2009; Kang et al., 2013), there was a lack of *in vivo* supporting evidence. Recently, Hsu et al. generated transgenic zebrafish lines to deplete miR-27b by expressing a miR-27b sponge (*C27bSP*) driven by the ubiquitous beta-actin promoter (*bC27bSP*) or the hepatocyte-specific *fabp10* promoter (*hC27bSP*). They demonstrated that the transgenic fish display hyperlipidemia, hepatic steatosis and increased white adipose tissue mass (Hsu et al., 2017), supporting a role of miR-27b in lipid metabolism *in vivo*. Another obesogenic model, *Tg(krt4Hsa.myrAkt1)*^*cy*18^, was initially generated to study skin cancer. However, the transgenic adults were found to be obese, with increased BMI, adipocyte hyperplasia, abnormal fat deposition, and glucose intolerance. These phenotypes likely result from ectopic expression of the constitutively active human AKT1 in several mesenchymal derived tissues (Chu et al., 2012). *Tg(krt4Hsa.myrAkt1)*^*cy*18^ fish differ from *Tg(b-actin:AgRP)* in two aspects. First*, Tg(krt4Hsa.myrAkt1)*^*cy*18^ fish do not display adipocyte hypertrophy. Second, *Tg(krt4Hsa.myrAkt1)*^*cy*18^ fish exhibit ectopic lipoma-like adipose tissue in dorsal muscle, gill arches, and tail bone tissues, whereas *Tg(b-actin:AgRP)* fish show a normal distribution of adipocyte tissues.

Multiple mutant lines have been identified that reveal genes and pathways contributing to lipid metabolism and adipose tissue regulation. These mutants are identified often because they have fatty liver at larval stages. Still other mutants are identified as adults due to increased adiposity. Although these mutants may share a common phenotype, they are due to the disruption of a diverse number of processes. Many mutants have larval hepatic steatosis, or fatty liver due to ER stress. In a “shelf screen” for liver size, *Foie gras*, and *cdipt*^*hi*559*Tg*/+^ were identified because they displayed fatty liver by 5 days of age (Sadler et al., 2005; Thakur et al., 2011). The affected gene product in *cdipt*^*hi*559*Tg*/+^, Cdipt, is necessary for phosphatidylinositol synthesis and lack of phosphatidylinositol causes ER stress and lipid accumulation in hepatocytes (Thakur et al., 2011). The *Foie gras* mutant results from mutation in transport protein particle 11 (*trappc 11*) that encodes a protein critical for ER to Golgi vesicular transport. As a result, the mutation causes pathogenic ER stress in hepatocytes, leading to fatty liver (DeRossi et al., 2016).

Beyond ER stress, other pathways leading to fatty liver have been identified using staining of never-fed mutant larvae with lipophilic dyes (Schlegel and Stainier, 2006; Kim et al., 2015; Hugo and Schlegel, 2017). One of these mutants, *harvest moon* (*hmn*), results from a mutation in glutamine-fructose-6-phosphate transamidase (*gfpt1*) gene (Hugo and Schlegel, 2017), while another mutant, 7466^mu110^, is caused by a mutation in vacuole membrane protein 1 (*vmp1*; Kim et al., 2015). These mutants feature lipid accumulation in hepatocytes and increased whole body adiposity and may provide clues to pathogenesis of fatty liver. Another mutant with fatty liver, *ducttrip* (*dtp*), was identified in a screen for mutations affecting the development of exocrine pancreas. The *dtp* mutant stems from a mutation in the gene encoding S-adenosylhomocysteine hydrolase and larvae exhibit mitochondrial dysfunction and liver degeneration in addition to hepatic steatosis and disrupted exocrine pancreas (Yee et al., 2005). While most fatty liver mutants do not survive to adulthood there are exceptions such as *red moon* (*rmn*) where both larvae and adults exhibit increased liver neutral lipids. The mutant is due to a loss-of-function of β-hydroxybutyrate transporter (*slc16a6a*; Hugo et al., 2012) and fatty liver results from the diversion of entrapped ketogenic precursor into lipids. Furthermore, the mutants are less tolerant of starvation. This mutant thus reveals a role of ketone body export in fasting energy homeostasis (Hugo et al., 2012). This diverse group of mutants highlights the complex regulation of lipid metabolism and how disruption at one node can lead to a phenotype of hepatic steatosis.

Other zebrafish mutants have alterations in adipose tissue. The zebrafish *vizzini* mutant exhibits decreased somatic growth and increased subcutaneous and visceral adipose tissues relative to body size. In *vizzini*, the subcutaneous adipose tissue (SAT) lipid droplets are extremely large although the number of lipid droplets in adipocytes is unchanged (McMenamin et al., 2013). This is due to a mutation in growth hormone 1 gene (*gh1*) leading to a premature stop codon. The phenotype is consistent with GH-deficient mice and humans that develop enlarged volume of SAT (Li et al., 1990; Wabitsch et al., 1995). Mutations in *cyp2r1* gene (Peng et al., 2017) also results in growth retardation and increased adiposity. These *cyp2r1* mutants are deficient in 1α,25(OH)_2_D_3_, the principal active form of vitamin D3, and 25(OH)D3 treatment rescues the growth and adiposity defects. In mammals, genetic and epidemiological data suggest a role of vitamin D deficiency in obesity, but vitamin D supplement fails to reduce the risk of obesity and associated pathologies (Rosen et al., 2012). These zebrafish mutants support a role of vitamin D in lipid metabolism and distribution. Mechanisms underlying distribution of adipose are critical as visceral fat is a better predictor than BMI of risk for cardiovascular diseases, insulin resistance and T2DM (Lebovitz and Banerji, 2005; InterAct et al., 2012). The loss of *plexin d1* function in zebrafish specifically impacts visceral fat (Minchin et al., 2015). PLEXIN D1 is one of the 67 genes identified in GWAS analyses in humans to be associated with visceral fat mass (Shungin et al., 2015), but the function of PLEXIN D1 in the distribution of fat mass was unknown. In zebrafish *plxnd1* mutants, visceral fat is reduced due to a decrease of lipid droplet size and adipocyte hyperplasia. Consequently, with high fat diet, the mutants preferentially store lipid in subcutaneous adipose tissue and are protected from developing insulin resistance (Minchin et al., 2015).

Taken together, these transgenic and mutant zebrafish lines further demonstrate conserved regulation of lipid metabolism. They also provide models in which to address mechanistic understanding of the underlying phenotypes.

### Utilities of zebrafish obesity models

One advantage of zebrafish models is the amenability for quick identification of chemical and genetic modifiers of the phenotype. The diet-induced obesity models have been used to test the effects of some dietary supplements on body fat accumulation. Green tea extract inhibited lipid accumulation (Tainaka et al., 2011; Meguro et al., 2015; Meguro and Hasumura, 2018), by decreasing the visceral adipose tissue volume and altering the expression of lipid catabolism genes (Hasumura et al., 2012). Eriocitrin, an antioxidative flavonoid in lemon, showed lipid-lowering effects in DIO zebrafish similar to that reported in a high-fat diet in rats (Hiramitsu et al., 2014). Oral administration of Yuzu (*Citrus junos* Siebold ex Tanaka) peel to DIO zebrafish exhibited anti-obesity effects by activating hepatic PPARα and adipocyte PPARγ pathways (Zang et al., 2014). Rhamnan sulfate, a sulphated polysaccharide from a marine green alga (*Monostroma nitidum*), also attenuated hepatic steatosis by suppressing lipogenesis (Zang et al., 2015a). Recently, a natural polyphenol, resveratrol, was reported to have anti-obesity effects via regulating lipid metabolism (Ran et al., 2017). Overall, DIO zebrafish is an attractive model system to evaluate the effects of functional foods and compounds on obesity development and treatment.

The diet-induced obesity models have also been used in drug testing. Tingaud-Sequeira et al. assessed the effect of small molecules on the whole-body adiposity after 24-h fasting in larvae previously overfed with egg yolk power (Tingaud-Sequeira et al., 2011). They found that two PPARγ agonists, rosiglitazone and TBT, a biocide found in antifouling paints, increases adiposity by inducing adipocyte hypertrophy and are thus obesogenic. In contrast, a PPARγ antagonist and an α1-adrenergic receptor agonist, known to promote lipolysis, are anti-obesogenic. Zhou et al. also performed proof of principle drug testing experiments using a similar model and found that all the 5 human hypolipidemic drugs exhibit significant hypolipidemic effect in zebrafish as they do in humans (Zhou et al., 2015). These results demonstrate the value of zebrafish obesity model on drug screening.

Genetic zebrafish models of obesity also provide mechanistic insights into the underlying causes. For instance, studies in the *cyp2r1* mutants that show increased adiposity and growth retardation identified *pgc1a* as a direct target for vitamin D receptor. As Pgc1a is a known master regulator of mitochondrial biogenesis, the study further showed that the increased adiposity results from impaired mitochondrial function (Peng et al., 2017). Similarly, using the *plxnd1* mutants, Minchin et al. investigated transcriptional changes in extracellular matrix genes (Minchin et al., 2015). They found that the mRNA and protein product of *col5a1* was increased and the visceral fat in the *plxnd1* mutants had more pronounced fibrillogenesis. Knocking down *col5a1* normalized the defects in visceral fat.

One cautionary note is that not all of the lipid metabolism genes are highly conserved in sequence and function in zebrafish. For example, the leptin protein of zebrafish is only 19% identical to the human protein. In mice and humans, leptin is an adipostatic hormone that regulates adipose mass, and failure of leptin signaling results in hyperphagia and obesity (Myers et al., 2010). Unlike mammals, leptin, and leptin receptor are not expressed in adipose tissue in zebrafish. Leptin receptor-deficient zebrafish primarily have disrupted glucose homeostasis (Michel et al., 2016), which is different from phenotypes observed in mouse models such as severe hyperphagia, hyperlipidemia and morbid obesity (Yen et al., 1976).

These examples illustrate the utility of zebrafish models for mechanistic investigations, drug testing and drug discovery in obesity and lipid metabolism. Thus far, the power these models remain largely untapped. It is anticipated that more mechanistic discoveries will be made from these and other zebrafish obesity models.

## Zebrafish diabetes models

### Pancreas development and glucose homeostasis in zebrafish

The morphogenesis and basic cellular architecture of zebrafish pancreas is similar to mammalian pancreas (Tehrani and Lin, 2011) with both exocrine and endocrine compartments. The endocrine compartment consists of glucagon-secreting α-cells, insulin-producing β-cells, somatostatin-producing δ-cells, ghrelin-producing ε-cells and pancreatic polypeptide producing PP-cells. These cells are arranged in a manner similar to mouse islets (Argenton et al., 1999; Biemar et al., 2001). The signaling pathways and mechanisms of zebrafish endocrine pancreas development are highly homologous to those of mammals (Kinkel and Prince, 2009). In addition to the pancreas, development and function of other organ systems involved in glucose homeostasis, including brain, liver, adipocyte tissue and skeletal muscle, are also conserved (Maddison and Chen, 2017). The conservation of the pancreas structure and glucose homeostasis system make zebrafish useful to identify novel targets in pancreas related diseases such as diabetes.

### Tools to studies glucose homeostasis in zebrafish

Numerous transgenic zebrafish strains with a fluorescent protein expression have been widely used to study pancreas development and glucose homeostasis in a whole living vertebrate (Kinkel and Prince, 2009; Tiso et al., 2009; Prince et al., 2017). For example, *Tg(-1.2ins:EGFP)* transgenic lines, where GFP expression is driven by the zebrafish preproinsulin promoter, provide a convenient fluorescent marker of β-cells (Xu et al., 2010) and insulin-expressing cells of the pancreatic islets can be visualized under a fluorescent microscope. Additionally, a transgenic line, *Tg(gcga:GFP)*, where GFP is driven by zebrafish preproglucagon promotor, marks pancreatic α-cell (Zecchin et al., 2007). Using these cell-specific transgenic lines, the β-cell and α-cell area and total numbers are easily measured to evaluate alterations in cell mass and number, which is a predictor for glucose clearance (Li et al., 2015; Maddison et al., 2015).

Methods for zebrafish pancreas function have been established, including fasting and postprandial glucose measurement, and intraperitoneal glucose tolerance tests as well as techniques for pancreas dissection and islet cell culture (Eames et al., 2010; Eames Nalle et al., 2017). In larvae, blood collection for glucose measurement is not a viable methodology but free glucose in whole larvae can be measured by a coupled-enzyme fluorescent assay (Jurczyk et al., 2011). For adult zebrafish, the small size (3–4 cm) makes blood collection challenging. Nevertheless, several protocols for blood collection have been developed, such as lateral incision in the region of the dorsal aorta, decapitation and tail ablation although these methods require sacrifice of the animal (Jagadeeswaran et al., 1999; Eames et al., 2010; Velasco-Santamaría et al., 2011). However, a method for repeated blood collection in the same individual adult zebrafish has been developed (Zang et al., 2013, 2015b). Blood glucose can be measured by hand-held glucose-meters (Eames et al., 2010; Zang et al., 2015b). Protocols for glucose tolerance test (GTT) have also been developed in zebrafish (Kinkel et al., 2010; Matsuda et al., 2017; Zang et al., 2017), which is the most used approach to diagnose diabetes mellitus or glucose intolerance in humans.

Measuring insulin and insulin function has presented more of a challenge. As surrogate indicators, *insulin* mRNA levels can be determined directly by qPCR (Michel et al., 2016) and insulin promoter activity may be determined indirectly by measuring EGFP signal intensity in *Tg(*−*1.0ins:EGFP)*^*sc*1^ zebrafish (Zang et al., 2017). An insulin antibody for immunostaining both in whole larvae or adult zebrafish histologic sections is also available (Kimmel et al., 2015). Semi-quantitative dot-blot has been used to compare insulin levels in different fish simultaneously (Olsen et al., 2012). But insulin release has yet to be reliably measured in zebrafish. GFP has been used to replace the C-peptide of proinsulin in a transgenic line as one method to measure insulin release (Eames et al., 2013). Phosphorylation of Akt has been used to assess insulin function as a method to investigate early stage insulin resistance (Maddison et al., 2015; Landgraf et al., 2017). Insulin sensitivity can also be assessed by intraperitoneal injection of insulin in hyperglycemic zebrafish (Capiotti et al., 2014; Maddison et al., 2015).

Much of the biology in glucose homeostasis, from genes to organs, is conserved from zebrafish to humans. The application of powerful live imaging in zebrafish, coupled with genetic, and chemical genetic manipulations, will likely yield insights to many outstanding questions in diabetes.

### Zebrafish diabetes models

Table 2 summaries zebrafish diabetes models developed by diet and genetic manipulations.

**Table 2:** Zebrafish diabetic models.

**Model type**		**Disease type**	**Treatment or genetic manipulation**	**Age**	**Phenotype**	**References**	**Disadvantage**
Induced models	Pancreatectomy	T1DM	physical removal of pancreas	Adult	Elevated blood glucose levels	Moss et al., 2009; Delaspre et al., 2015	Technically difficult
	Chemical-ablation of β-cells	T1DM	Intraperitoneal injection of streptozotocin (STZ)	Adult	Hyperglycemia and diabetic complications	Moss et al., 2009; Olsen et al., 2010; Intine et al., 2013	Rapid recovery
		T1DM	Alloxane exposure through incubation or IP injection	Larvae, adult	β-cell necrosis, decreased neuromast number,	Moss et al., 2009; Nam et al., 2015; Castañeda et al., 2017	Rapid recovery
	Glucose immersion	T2DM	Incubation in glucose solution	Adult	Hyperglycaemia, impaired response to inuslin, diabetic retinopathy	Gleeson et al., 2007; Alvarez et al., 2010; Capiotti et al., 2014; Connaughton et al., 2016	Requires frequent solution exchange
	Over-nutrition	T2DM & Obesity	Overfeeding zebrafish with a commercial food	Adult	Hyperglycaemia, glucose intolerance, insulin resistance	Zang et al., 2017	
Targeted genetic ablation	NTR-mediated cell ablation	T1DM	Nitroreductase (NTR) expressing transgenic lines exposed to metronidazole (MTZ) through incubation or IP injection	Larvae, adult	Destroyed islet tissue, increased blood glucose levels	Curado et al., 2007, 2008; Pisharath et al., 2007; Moss et al., 2009; Ninov et al., 2013; Delaspre et al., 2015; Ye et al., 2015	Rapid recovery
	Induction of apoptosis through expression of activated Bid	T1DM	*Tg(1.2ins:htBid^*TE*−*ON*^; LR)* Induced by doxycycline and tebufenozide	Larvae	β-cell ablation, increased free glucose levels	Li et al., 2014	Rapid recovery
Transgenic lines	*Tg(acta1:dnIGF1R-EGFP)*	T2DM	Transgenic expression of a dominant-negative IGF-I receptor (IGF-IR) in skeletal muscle	Adult	Increased fasting blood glucose level	Zang et al., 2017	
	*Tg(actb2:cas9; (U6x:sgRNA(insra/b)*	T2DM	Liver specific knockdown of the insulin receptor a and b	Larvae	Hyperglycemia, insulin resistance	Yin et al., 2015	
	*Tg(C43G-GFP)*	MODY 10	Transgenic expression of C43G human proinsulin	Larvae, adult	Normal glucose homeostasis, no loss in β-cell mass	Eames et al., 2013	
Mutant lines	*liger^*s*430^ (hnf1ba^*s*430^)*	MODY5	Mutation in *hnf1ba*	Larvae	MODY5-like pancreas hypoplasia, reduced β-cell numbers	Lancman et al., 2013	
	CRISPR induced gene deficiency	MODY6	Mutation in *neurod1*	Larvae	Failed endocrine cell differentiation, increased free glucose levels	Dalgin and Prince, 2015	
	*pdx1^*sa*280^*	MODY4	Mutation in *pdx1*	Larvae, adult	Reduced β-cell numbers, disrupted glucose homeostasis, sensitivity to overnutrition	Kimmel et al., 2015	

#### Type 1 diabetes mellitus models

Type 1 diabetes mellitus (T1DM) is primarily an autoimmune disease caused by destruction of insulin producing pancreatic β-cells. Although an autoimmune derived T1DM model is lacking in zebrafish, several models have been developed using targeted β-cell damage. Three methods of β-cell destruction have been applied: surgical removal, chemical-dependent ablation, and genetic ablation. Pancreatectomy is feasible under the microscope in transgenic zebrafish with islet specific expression of GFP (Moss et al., 2009; Delaspre et al., 2015). However, this method is technically difficult and is not commonly used in zebrafish. Chemical-induced diabetes is widely used in rodents and also in zebrafish. Intraperitoneal injection of streptozotocin (STZ) is effective at β-cell ablation in adult zebrafish and eventually causes elevated fasting blood glucose and reduced insulin levels (Moss et al., 2009; Olsen et al., 2010; Intine et al., 2013). A total of 6 administrations of STZ within 4 weeks induces stable hyperglycemia and diabetic complications including retinopathy, nephropathy, and impaired fin regeneration. Alloxan can also selectively kill β-cells in zebrafish larvae (Nam et al., 2015; Castañeda et al., 2017). However, these compounds also exhibit other toxicity. Multiple genetic model of T1DM have been reported. Although stable expression of cell-lethal diphtheria toxin A chain (DTA) can eliminate all β-cells, these fish have growth retardation and fail to thrive (Ninov et al., 2013). Therefore, inducible β-cell ablation has been the preferred method for modeling T1DM. Two approaches of inducible β-cell ablation have been reported. In one approach, transgenic zebrafish lines with β-cell specific expression of the bacterial nitroreductase (NTR) enzyme, are exposed to the prodrug metronidazole (MTZ), the NTR substrate, which is converted into a cytotoxic compound that rapidly induces β-cell apoptosis (Curado et al., 2007, 2008; Pisharath et al., 2007; Ye et al., 2015). This NTR/MTZ ablation system is used for β-cell regeneration research as the elimination of β-cell occurs in 18–24 h after MTZ administration and recovers within 3–4 days after MTZ washout. A different approach has been to use a combinatorial, inducible transgene where the insulin promoter drives the expression of a doxycycline/ecdysone-dependent transcription factor and the TetOR-based promoter to express activated human Bid that triggers apoptosis (Li et al., 2014). Ablation models all face the same hurdle in that zebrafish have a remarkable regenerative capacity and β-cell mass is restored once the ablation mechanism is removed.

#### Type 2 diabetes mellitus models

T2DM is characterized by insulin resistance and β-cell dysfunction. β-cell death may also occur in long standing T2DM. Both nutritional and genetic approaches have been used to generate T2DM models in zebrafish. Immersion of zebrafish in glucose solution is a widely-used method because of its convenience. Immersing adult zebrafish into alternating concentrations of 0 and 2% glucose every other day for 28–30 days, or chronic exposure to 2% glucose solution for 14 days, induces diabetic phenotypes, including elevated blood glucose levels and impaired response to exogenous insulin (Gleeson et al., 2007; Alvarez et al., 2010; Capiotti et al., 2014), similar to mice following 6 weeks of high-galactose diet (Joussen et al., 2009). Young zebrafish (4–11 months) acclimate to glucose exposure better than older zebrafish (1–3 years), but persistent hyperglycemia, can be achieved even in young zebrafish by gradually increasing the glucose concentration (Connaughton et al., 2016).

Obesity is the major risk factor for T2DM. High-fat diet causes both obesity and T2DM in rodent models (Winzell and Ahrén, 2004). In zebrafish, overfeeding with a commercial food quickly caused insulin resistance, elevated fasting blood glucose, and impaired glucose tolerance (Zang et al., 2017). Calorie restriction and anti-diabetic drugs (metformin and glimepiride) ameliorated the hyperglycemia in the overfed zebrafish. These drugs are both frequently prescribed treatments for T2DM and their effectiveness in the zebrafish model demonstrates conservation in glucose homeostasis pathways.

Insulin resistance is a major driver of T2DM. Our lab has developed two transgenic models of insulin resistance. In one model, skeletal muscle insulin resistance is achieved by transgenic expression of a dominant-negative IGF-I receptor (IGF-IR) in skeletal muscle. The transgenic fish showed impaired Akt phosphorylation postprandially or after insulin administration (Maddison et al., 2015). These fish had significantly increased fasting blood glucose as early as 3-month old compared to wild-type fish and is exacerbated by overfeeding (unpublished data). In the other model, insulin resistance is achieved through liver specific knockdown of the insulin receptors using CRISPR/Cas9 (Yin et al., 2015). Similar to mice and humans, liver insulin resistance causes fasting hypoglycemia and postprandial hyperglycemia. Since muscle and liver insulin resistance are thought to be the major drivers of T2DM, these models will be useful to dissect the progression of T2DM.

Another type of diabetes, MODY (maturity-onset diabetes of the young), is a rare, autosomal dominant, noninsulin-dependent and monogenic form of diabetes resulting from pancreatic β-cell dysfunction with an onset before 25 years of age. Since this disease is caused by mutation in a single gene, with different genes leading to different forms, MODY models can be developed by targeted gene ablation. However, as in mice, the mode of inheritance in MODY gene mutations is usually recessive, not autosomal dominant. MODY5 stems from mutations in hepatocyte nuclear factor 1β (HNF1β). A zebrafish *hnf1ba* mutant line (*hnf1ba*^*s*430^) was identified from a zebrafish ENU mutagenesis screen (Lancman et al., 2013). The homozygous mutants exhibit pancreas hypoplasia and reduced β-cell numbers similar to MODY5. MODY6 results from mutations in NEUROD1 (Malecki et al., 1999). In mice, disruption of *NeuroD1* leads to diabetes and premature death (Naya et al., 1997). In zebrafish *neurod1* deficiency led to failed endocrine cell differentiation and increased free glucose levels in larvae (Dalgin and Prince, 2015). MODY4 is a result of PDX1 mutation (Stoffers et al., 1997) and a *pdx1* mutant line exhibited reduced β-cell numbers, disrupted glucose homeostasis, sensitivity to over-nutrition and is responsive to anti-diabetic drug treatment (Kimmel et al., 2015). The adult *pdx1* mutant zebrafish have small body size and decreased viability. MODY10 results from mutations in INS gene (Meur et al., 2010). A transgenic line expressing a mutated preproinsulin protein (C43G) has been developed (Eames et al., 2013). Interestingly, glucose homeostasis and β-cell mass were not altered in these fish, even though excess proinsulin accumulates in endoplasmic reticulum (ER). This could be due to the regenerative capacity of the zebrafish leading to turnover of the dysfunctional β-cells. However, this provides an opportunity to investigate misfolded proinsulin and ER stress in a non-diabetic *in vivo* system. Together, these MODY models develop phenotypes observed in patients, further supporting the utility of zebrafish as a diabetes model.

Although an appropriate model is still lacking for studying the long-term effect of diabetes, there have been approaches to study diabetic complications. For example, long immersion of larval or adult zebrafish in glucose solution has been used to model chronic hyperglycemia (Capiotti et al., 2014; Connaughton et al., 2016). This approach has been used to study diabetic retinopathy (Gleeson et al., 2007; Jung et al., 2016) as well as changes in bone metabolism (Carnovali et al., 2016). Inducing hyperglycemia through repeated STZ treatment in adult fish can impair wound healing (Olsen et al., 2010) and can cause heritable epigenetic changes after normalization of glycemia (Olsen et al., 2012). These studies underscore the lasting consequences of disrupting glucose control in zebrafish.

Overall, zebrafish offers particular advantages to the study of metabolic diseases. Models for studying obesity, pancreas regeneration, hyperglycemia, and diabetic complications have been established and will promote the understanding of the disease mechanisms, and provide new targets for disease therapy.

## Author contributions

All authors listed have made a substantial, direct and intellectual contribution to the work, and approved it for publication.

### Conflict of interest statement

The authors declare that the research was conducted in the absence of any commercial or financial relationships that could be construed as a potential conflict of interest.
